# Increasing motivation and well-being among medical students using curricular self-experience group sessions—a randomized controlled trial

**DOI:** 10.1080/10872981.2025.2519386

**Published:** 2025-06-13

**Authors:** Philipp Spitzer, Claire Mittmann, Janine Utz, Stefan Mestermann, Teresa Festl-Wietek, Johannes Kornhuber, Anne Herrmann-Werner

**Affiliations:** a Department of Psychiatry and Psychotherapy, University Hospital Erlangen, Friedrich-Alexander-Universität Erlangen-Nürnberg, Erlangen, Germany; b Tübingen Institute for Medical Education (TIME), University of Tübingen, Tübingen, Germany; c Department of Internal Medicine VI - Psychosomatic Medicine and Psychotherapy, University of Tübingen, Tübingen, Germany

**Keywords:** Medical education, self-experience, mental health, motivation, self-determination, motivational interviewing, group therapy

## Abstract

**Background:**

Medical students often face increased stress, reduced well-being, and decreased intrinsic motivation. Deci and Ryan’s self-determination theory suggests that intrinsic motivation can be increased through autonomy, relatedness and competence. Intrinsic motivation and relaxation techniques have been shown to reduce stress. Therefore, this pilot study investigates whether promoting relatedness, autonomy and competence through participation in self-experience group therapy increases intrinsic motivation and reduces stress in medical students.

**Methods:**

A longitudinal randomized controlled trial was conducted with 139 medical students. The students were participants in a one-week psychiatry internship in the winter semester of 2023–4. They were randomly assigned to either a daily relaxation group or self-experience group therapy based on motivational interviewing. Data were collected from pre- and post-internship groups as well as several weeks later using the Self-Regulation Questionnaire for Learning, the Intrinsic Motivation Inventory, the Perceived Stress Scale, the Visual Analog Scale for Stress, and the WHO-5 Scale for Well-Being. Focus group interviews were conducted with students and therapists and were thematically analyzed. The students’ performance was compared and analyzed.

**Results:**

The students in both groups exhibited increased autonomous regulation with respect to learning at the end of the internship (*p* < .001, *d* = 3.29) and improved well-being (*p* < .001, *d* = .275) as evaluated via repeated-measures ANOVA. Cross-sectional testing with at test revealed that participants in the self-experience group sessions presented higher levels of relatedness (*p* = .005, *d* = .505) and intrinsic motivation (*p* = .002, *d* = .542) than participants in the relaxation group. The self-experience group sessions were well accepted by the students, who preferred these sessions to the relaxation group. No differences were detected at the end of the semester.

**Conclusion:**

The integration of self-experience therapy into student teaching is a suitable way of eliciting short-term feelings of relatedness and increasing students’ motivation to learn while simultaneously reducing their experiences of stress over a one-week period.

## Introduction

Stress, burnout, and stress-related illnesses such as depression and anxiety are increasingly prevalent among medical students [1–4]. Educational scientists have called for the development of evidence-based programs with the aim of improving students’ mental health and incorporating these programs into relevant curricula [5]. While the burden of stress increases, students’ motivation for learning decreases [6–8]. Because intrinsic motivation seems to be negatively correlated with the experience of stress, a powerful way to restore students’ mental health may be to promote intrinsic motivation. However, no such interventions exist. We developed self-experience group sessions based on the principles of motivational interviewing [9] and evaluated their effects in a randomized controlled trial with an active control group that practiced relaxation techniques.

According to Deci and Ryan’s self-determination theory, motivation can be measured on a spectrum of extrinsic to intrinsic motivation [10]. While students who predominantly experience extrinsic motivation study primarily to satisfy, for example, their parents, students who are intrinsically motivated study primarily because of their own interest and their satisfaction with the learning material. This theory does not view motivation as a rigid personality trait but rather as a variable that is dependent on several influencing factors [11]. According to Deci and Ryan, intrinsic motivation can be promoted particularly through the experience of autonomy, the experience of competence and a feeling of relatedness [10].

**I**ncreases in intrinsic motivation appear to be associated with reduced experiences of stress [12,13]. In a previous study, our group succeeded in promoting intrinsic motivation and reducing stress among medical students by optimizing an existing practical training program in light of the basic motivational needs identified by Deci and Ryan [14]. In that study, we found that even subtle changes in the course program, such as introducing group games during breaks, providing only ‘positive’ resource-oriented feedback, and establishing check-in and check-out rounds at the beginning and end of the day, were effective means of reducing stress [14]. Positive, resource-oriented feedback means that feedback was given only to reinforce the students’ strengths. However, the study did not examine long-term effects or effects on well-being. In addition, the intervention led to lower exam scores. This finding is of particular interest because self-determination theory suggests that greater intrinsic motivation is associated with better learning outcomes [15–18]. Well-established individual-focused alternatives that can be used to reduce stress and anxiety include relaxation techniques such as autogeneous training and muscle relaxation using the model developed by Jacobson and meditation [19,20]. For medical students, yoga, autogenic training and muscle relaxation have been reported to be effective ways to reduce stress [21–23]. However, the effect sizes of these individual-focused approaches are only small to medium, and interventions that focus on the learning environment seem more promising [24].

We know from the training of psychotherapists, which include self-experience sessions as a compulsory component, that such sessions have the potential to increase interpersonal skills, knowledge and well-being [25]. In the context of medical students, researchers have also reported that reflection groups can improve participants’ empathy while simultaneously reducing symptoms of burnout [26–29]. The learning objectives stipulated in Chapter VIII.6 of the German national competence-based catalog of learning objectives in medicine (NKLM) include competences pertaining to self-reflection and self-care [30]. These learning objectives can integrate learning units that allow students to reflect on their motives and needs into the relevant curricula [31,32]. In addition to the benefits to students as future doctors, it appears that patients also benefit from students’ self-experience training [33].

We conducted a pilot study to determine whether it is feasible to integrate self-experience group therapy sessions into the medical curriculum and whether participation in these groups can elicit feelings of relatedness on the part of students, which can promote their intrinsic motivation for learning while reducing their perceived stress.

We hypothesize that compared with relaxation training, even short-term exposure to self-experience therapy sessions results in stronger feelings of relatedness, increased intrinsic motivation and reduced stress.

## Methods

### Research design and participants

A longitudinal randomized controlled study was conducted at Friedrich Alexander University Erlangen-Nuremberg during the winter semester of 2023–24 to investigate the effects of self-experience sessions on intrinsic motivation, stress and well-being among 4^th^-year medical students. All students in the psychiatry internship were invited to participate in this study. Only students who did not speak German or who did not have the means to complete the online questionnaires were excluded from the study. Of the 170 students who took the course during this term, 139 (83%, m: 39, f: 100; mean age: 25.3 ± 3,0) participated in the study and completed the required online questionnaires. Only participants who fully completed the questionnaire were included in the final analysis. For the participants’ characteristics, please see Table 1.

**Table 1.** Student participation in the experimental groups.

	Self-experience	Relaxation
Number of participants [n] total male female	70 23 (32.9%) 47 (67.1%)	69 16 (23.2%) 53 (76.8%)
Study progress [n] 8. semester 9. semester 10. semester 11. semester 12. semester	62 (88.6%) 7 (10.0%) 1 (1.43%) 0 0	55 (79.7%) 10 (14.5%) 2 (2.90%) 0 2 (2.90%)
Age [years] (mean ± SD)	25,2 ± 3,5	25,1 ± 2,8

Number of participants, their gender and study progress as well as their mean age (±SD) in each group.

With respect to sample size, 70 participants were needed for each group to detect medium-sized effects (d = 0.5) with a probability of 90% and an α-error of 0.05.

Students in the internship were permitted to select one of 14 time points at which the internship was offered. At each time point, the participating students were randomly assigned to one of two groups, each of which consisted of six to seven students. One group was the self-experience group, whereas the other group was the relaxation group, which was held in parallel with the psychiatry internship. Both groups were aware of each other’s existence, so blinding was impossible. All groups of both types were led by one of three trained therapists.

Prior to and following the course as well as at the end of the corresponding semester, the students were invited to complete an online survey. The learning outcome was evaluated via an objective structured clinical examination (OSCE) that was administered on Monday following the week of the course. The students’ responses at each time point and their scores on the OSCE at the end of the course were aligned using a coding system. For each participating student, this code consisted of the first and last letter of the student’s mother’s first name, the date of the student’s mother’s birthday and the first and last letter of the student’s town of birth. This code was known only to the individual participating student and could not be tracked.

### Intervention

The intervention was integrated into our internship in psychiatry [14,34]. The internship consisted of five consecutive afternoons of practical training (Monday to Friday, 4–5 hours daily, totaling 21 hours) at our psychiatric clinic followed by an OSCE the next Monday. Before the internship, the students completed a 10-hour self-paced multimedia online course covering theoretical basics.

During onsite training, each student interviewed both real and simulated patients and received feedback from simulated patients, peers, and the lecturer. In the OSCE, students were assessed on history-taking, communication skills, and case reporting using a standardized patient scenario. The two groups, each of which consisted of six to seven students, were randomly assigned to participate in either five 60-minute self-experience group sessions or the same number of relaxation sessions; the latter served as the control condition.

#### Self-experience group therapy

The manual used for the self-experience group sessions was developed by the authors of this study and is available on request. Some of the authors are experienced group psychotherapists and experts in motivational interviewing. The approach used in the manual was based on the theory of motivational interviewing. **T**hese sessions were designed to increase the participants’ intrinsic motivation to become involved in learning activities. Motivational interviewing is a nondirective counseling technique that was developed by Miller and Rollnick in 1991 with the goal of supporting patients suffering from addiction [9]. Since that time, motivational interviewing has become increasingly important in other disciplines as well as in the field of prevention [35,36]. Adaptations of this approach to group therapy have been published [37]. A large body of evidence indicates that motivational interviewing can help promote intrinsic motivation in various contexts [38,39].

The initial session included in our manual focused primarily on establishing rapport between the therapist and the group as well as among the participants in the group. This was achieved by asking the students to choose pictures from a pile of motif cards and to use these pictures to reflect on their state of mind during their studies. In the second session, the participants engaged in an exploration of their personal values in the context of their academic and personal pursuits. The students chose their five most important values from a selection of over 50 personal value cards and participated in group discussions to understand where in their studies these values were realized. In the third session, the group facilitated the identification of each participant’s personal strengths by implementing an ‘ability shower’, in which all group members told each other what characteristics and competencies they valued in other group members. In the fourth session, the students identified their position within the group according to Schindler’s rank-dynamic model, which was developed in 1957 [40]. The purpose of this exercise was to recognize and reflect on their position in groups (e.g., leader, expert, follower, outsider, doubter). The objective of the fifth and final session was to develop an action plan for the integration of self-realization into the students’ personal life. This plan was intended to help the participants implement ideas for change that emerged from the previous meetings.

#### Relaxation group

A customized manual was created for the relaxation group. On the initial day of the program, the group participated in two sessions involving autogenic training. Autogenic training is a relaxation technique that uses self-suggestion and visualization to promote physical and mental calmness by focusing on sensations such as warmth and heaviness in the body. On the second day, the group was taught and practiced progressive muscle relaxation. On the third day, the group participated in a body scan, which served as an illustrative example of a relaxation technique rooted in mindfulness-based therapy. On the fourth day, the group engaged in two dream journeys, and on the final day, they focused on training in various breathing techniques.

### Training of therapists

All groups were led by one of three therapists. One was a senior physician, a specialist in psychiatry and psychotherapy and an experienced group therapist with 15 years of professional experience. The other two were junior doctors in their second and third years of training as specialists in psychiatry and psychotherapy. The senior doctor trained and supervised the others in the interventions.

### Questionnaires and data acquisition

**Figure 1.**
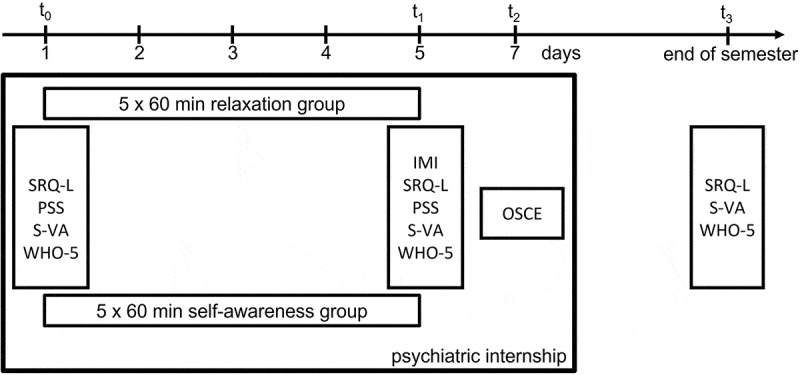
Study design.

SRQ-L: Self-Regulation Questionnaire for Learning, PSS: Perceived Stress Scale; S-VA: Stress – Visual Analog Scale; WHO-5: Well-being Questionnaire of the World Health Organization; IMI: Intrinsic Motivation Inventory; OSCE: Objective Structured Clinical Examination

The timeline for each individual group is illustrated in Figure 1. At the beginning of the practical psychiatry course, the students were invited to complete an online survey that included several well-established and validated self-assessment questionnaires: the Self-Regulation Questionnaire for Learning (SRQ-L), the WHO-5 Scale for Well-Being, the Perceived Stress Scale (PSS) and a visual analog scale for stress (S-VA).

At the end of the fifth day, the students were once again invited to complete an online survey that included these four questionnaires (SRQ-L, PSS, S-VA and WHO-5) and a task evaluation questionnaire that included the interest/enjoyment, pressure, choice and relatedness subscales of the Intrinsic Motivation Scale as well as the relatedness subscale of the Intrinsic Motivation Inventory (IMI) [41,42]. Furthermore, after each internship, a group interview that included all the students from both study groups was conducted. In this focus group interview, students were invited to identify the elements of the sessions that they would maintain and to propose improvements. The interviews were recorded in written form.

An OSCE was subsequently scheduled for the Monday following the course with the goal of assessing the learning outcomes of this practical course in psychiatry.

After the semester, the students were invited to complete a final survey that included the SRQ-L, S-VA and WHO-5 questionnaires.

The participants’ responses to the individual questionnaires and the results of the OSCE were individually correlated using the coding system detailed above. After the winter semester, the three therapists were interviewed to collect their experiences with the group therapies.

#### Self-regulation questionnaire for learning (SRQ-L)

We assessed students’ motivation for pursuing their studies via the Self-Regulation Questionnaire for Learning (SRQ-L) [43]. This questionnaire includes 14 items and two scales that measure autonomous regulation and controlled regulation, which represent the two extremes of motivation; each was scored using a 7-point Likert scale [43]. The students were required to score items such as ‘I will participate actively in the psychiatry internship because learning to interview well is an important part of becoming a doctor’ and ‘I will participate actively in the psychiatry internship because others would think badly of me if I didn’t’ on a 7-point scale ranging from “not at all true: (1 point) to ‘somewhat true’ (4 points) to ‘very true’ (7 points). In previous studies, the alpha coefficients for these two subscales were approximately 0.75 for controlled regulation and 0.80 for autonomous regulation. In this study, we found a Cronbach’s alpha of 0.76 for controlled regulation and 0.86 for autonomous regulation. Validation was performed at the level of these two categories [32]. This questionnaire enabled the calculation of a ‘relative autonomy index’ by subtracting the participants’ scores on the controlled regulation subscale from the scores on the autonomous regulation subscale [32].

#### Perceived stress scale

Stress was assessed via the German version of the Perceived Stress Scale (PSS) [44] and a visual analog scale (S-VA) [45,46]. The PSS contains ten items, such as, ‘In the last week, how often have you been upset because of something that happened unexpectedly?’ and “In the last week, how often have you felt nervous and ‘stressed?’ The German version of the PSS contains two subscales: helplessness and self-efficacy [44]. In our sample, the full PSS scale showed good reliability with a Cronbach’s alpha of 0.88. The Cronbach’s alpha was 0.83 for the helplessness subscale and 0.76 for the self-efficacy scale.

#### The WHO-5 scale for well-being

The WHO-5 scale is a widely used screening tool to measure mental well-being [47,48]. The scale includes five items, such as ‘I have felt calm and relaxed’ and ‘I have felt active and vigorous’. Participants respond to these items on a six-point scale ranging from ‘at no time’ to ‘all the time’. In our sample, the questionnaire showed good reliability with a Cronbach’s alpha of 0.79.

#### Intrinsic motivation inventory (IMI)

We used a task evaluation questionnaire that included the interest/enjoyment, pressure and choice subscales of the Intrinsic Motivation Scale as well as the relatedness subscale of the Intrinsic Motivation Inventory (IMI) [41,42]. The interest/enjoyment subscale includes items such as ‘While I was working on the task, I was thinking about how much I enjoyed it’ and represents a self-reported measure of intrinsic motivation (Cronbach’s alpha 0.87). The perceived competence scale (e.g., ‘I think I am pretty good at this task’) and the perceived choice scale (e.g., ‘I felt that it was my choice to do the task’), which reflect a sense of autonomy (Cronbach’s alpha 0.84), as well as the scales for pressure/tension (e.g., ‘I felt tense while doing the task’) (Cronbach’s alpha 0.85) and relatedness (e.g., ‘I felt like I could really trust my group’) (Cronbach’s alpha 0.84), are viewed as predictors of intrinsic motivation. Each of the 30 items included in the IMI is scored on a 7-point Likert scale that ranges from ‘not at all true’ (1 point) to ‘somewhat true’ (4 points) to ‘very true’ (7 points). Strong support for the validity of the IMI has been reported, as has evidence indicating the adequate reliability of its subscales [42].

### Data analysis

The dataset was analyzed via the statistical software packages SPSS (SPSS® 29, IBM®, Armonk, USA) and Prism v6.07 (GraphPad®, San Diego, USA). Figures were prepared with Prism v6.07 (GraphPad®, San Diego, USA). The assumptions of linearity, normality, and homoscedasticity were evaluated to determine the suitability of the models. Only complete questionnaires were included in the analysis. No data points were removed. Chi-square tests were used to assess differences in nominally scaled variables. T tests for independent or dependent samples were conducted for cross-sectional comparisons of variables that were collected at only one point in time. Welch’s correction was used for heteroscedastic samples. To evaluate the impacts of the intervention on variables collected before and after the internship, two-way analyses of variance for repeated measures (ANOVAs) were conducted [49]. The data collected at the end of the semester were analyzed separately with t tests because the values were collected during the students’ examination phase and therefore differed considerably from the values recorded during the internship. The effect sizes were calculated and interpreted in line with the suggestions of Cohen [50]. A chi-square test for association was conducted to compare categorical variables. Correlations were calculated via the Pearson correlation coefficient. The results were determined to be significant when *p* < .05. A *p* value between .05 and .1 indicated a trend. Focus group interviews were evaluated via thematic analysis [51]. First, notes taken during the interviews were coded, and initial themes were generated. The themes were subsequently reviewed in light of the initial codes and the entire dataset. Finally, the themes were defined more specifically, and a semiquantitative readout was produced.

### Ethics

All students who participated in the online surveys did so voluntarily and were explicitly informed that their participation would not impact their examination grades and that the data they provided would be pseudonymized according to the coding system detailed above. Consent to participate in the study was provided online when the students completed the questionnaires. No data that enabled the recognition of individual students were collected or reported. The procedures used in this study were approved by the ethics committee of the Friedrich-Alexander University Erlangen-Nuremberg (**r**eference number 23–306-S).

## Results

### Participant characteristics

A chi-square test was used to assess differences in gender and study progress within the study groups. No expected cell frequencies were below 5. The results revealed no significant association between gender and participation in the self-experience group (χ^2^ (1) = 1.61, *p* = .205, *V* = .108) or between study progress and participation in the self-experience group (χ^2^ (3) = 3.28, *p* = .351, *V* = .153). Additionally, age did not differ between the groups as assessed via a t test (*t*_(139)_ = −3.93, *p* = .858, *d* = .030) (Table 1).

### Feelings of relatedness and intrinsic motivation after participation in self-experience groups

Among the 170 students who participated in the practical course in psychiatry, 139 (82%) completed the questionnaires both before and after the course. Among these students, 69 (49.6%) participated in the relaxation group, whereas 70 (50.4%) participated in the self-experience group. No data points were removed.

**Figure 2.**
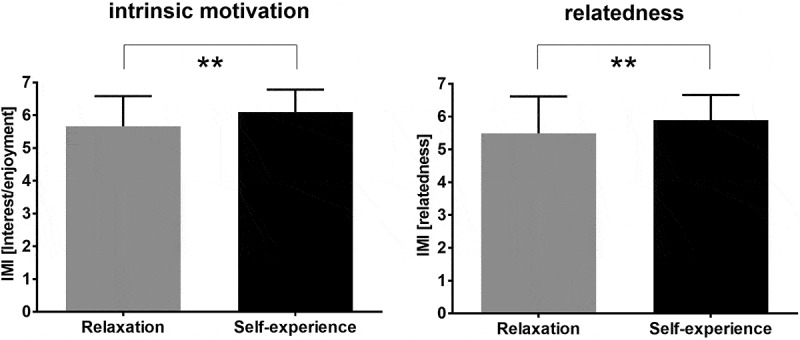
Intrinsic motivation and feelings of relatedness at the end of the intervention as assessed via the intrinsic motivation inventory (IMI).

At the end of the course, students’ intrinsic motivation and their feelings of relatedness, pressure, autonomy and competence were evaluated via the IMI. The students in the self-experience group presented higher levels of intrinsic motivation (5.66 ± .922 vs. 6.11 ± .701; *t*_(139)_ = −3.10, *p* = .002, *d* = .542) and more intense feelings of relatedness (5.49 ± 1.12 vs. 5.97 ± .719, *t*_(129)_ = −2.89, *p* = .005, *d* = .505) than the students in the relaxation group did (Figure 2). No changes were observed in terms of feelings of pressure, autonomy or competence.

** *p* < .01

### Differences in well-being between the two study groups

**Figure 3.**
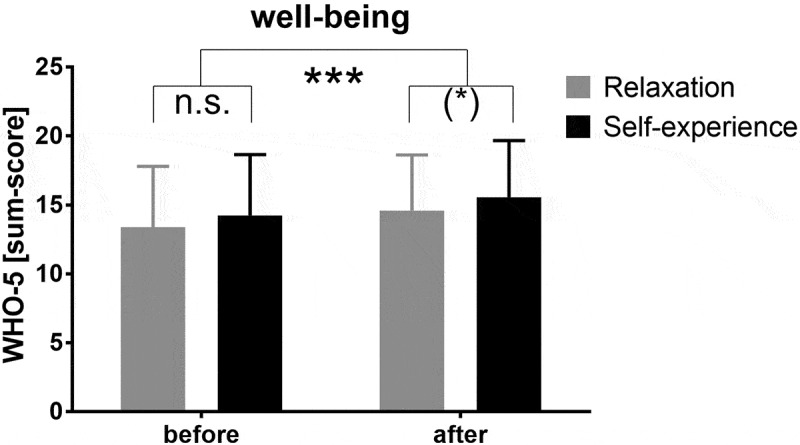
Well-being as assessed via the WHO-5 questionnaire before and after the intervention.

Students’ well-being was assessed via the WHO-5 questionnaire both before and after the internship. Two-way ANOVA revealed highly significantly increased well-being in both study groups following the course (*F*_(1, 126)_ = 11.5, *p* < .001, *d* = .275). The proportion of students who obtained a WHO-5 score of seven points or below decreased from 10.7% to 3.3% within the overall study cohort. Additionally, a marginally nonsignificant improvement in well-being was observed in the self-experience group compared with the relaxation group (*F*_(1, 126)_ = 2.99, *p* = .086, *d* = .261) (Figure 3). Well-being was significantly correlated with intrinsic motivation (*r* = .496, *p* < .001). No changes over time or group effects were observed with respect to stress as measured via the PSS or the visual analog scale. However, stress was negatively correlated with well-being (PSS *r* = −.593, *p* < .001; S-VA *r* = −.552, *p* < .001) and intrinsic motivation (PSS *r* = −.147, *p* = .079; S-VA *r* = −.275, *p* < .001).

(*) *p* < .1, *** *p* < .001

### Differences in autonomous regulation

**Figure 4.**
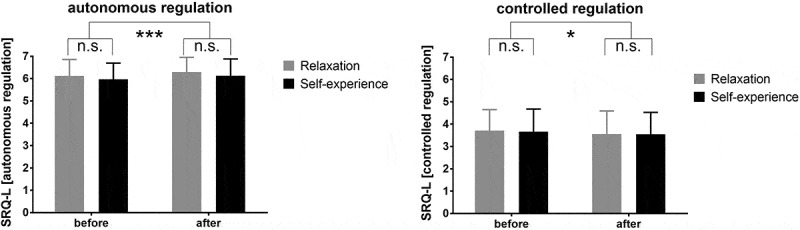
Source of motivation before and after the intervention as assessed via the self-regulation questionnaire for learning (SRQ-L).

Autonomous and controlled regulation were measured via the SRQ-L. The autonomous regulation exhibited by students in both groups increased over time (*F*_(1, 126)_ = 13.7, *p* < .001, *d* = 3.29), whereas their controlled regulation decreased (*F*_(1, 126)_ = 5.87, *p* = .017, *d* = 2.15). No group effects were observed for any of the parameters (Figure 4). In line with our expectations, autonomous regulation was correlated with intrinsic motivation (*r* = .525, *p* < .001), whereas controlled regulation was correlated with stress (PSS *r* = .245, *p* = .003, S-VA *r* = .192, *p* = .018).

* *p* < .05, *** *p* < .001

### Differences in learning outcomes

Following the practical training in psychiatry, learning was assessed via an OSCE that included three tasks (general history-taking, psychopathological examination, and patient handover). The students in the relaxation group achieved scores of 47.1 ± 6.7 points out of 60, whereas students in the self-experience group achieved scores of 47.7 ± 4.4 points. No significant differences between the study groups were observed with respect to the OSCE (*t*_(126)_ = −.572, *p* = .569, *d* = .102).

### Autonomous regulation, stress and well-being at the end of the semester

At the end of the semester, stress, well-being and the relative autonomy index were assessed again while the students waited to take their final exam in psychiatry. A total of 73 (42.9%) students also completed the questionnaires at the end of the semester (35 students (47.9%) in the relaxation group and 38 students (52.1%) in the self-experience group). No significant differences between the study groups were observed with respect to these parameters.

### Focus group discussions with students and therapists

Themes were manually generated and reviewed by two of the authors after the students’ statements were coded for content. Repeated codes were counted to produce a semiquantitative readout. The emerging themes were (1) emotional closeness within the group, (2) personal development, (3) the effect of the intervention on group dynamics, (4) the general rating of the intervention, (5) organizational aspects and (6) difficulties with specific aspects of the intervention.

1. Emotional closeness within the group: Emotional intimacy was described as ‘appropriate’ by the students in all focus groups.
2. Personal development: While the relaxation group was described by students in seven out of nine focus groups as a form of teaching that offered little added value, participation in the self-experience group was described by students in six out of nine groups as helpful for their personal development.
3. Effect of the intervention on group dynamics: Students in six out of nine groups described their participation in the self-experience group as beneficial with respect to group dynamics during the internship. Students in only one group described the dynamics as ‘sluggish’.
4. General rating of the intervention: While students in all focus groups reported that the self-experience group was ‘very good’, they were less convinced of the added value of the relaxation group, which was reported to be ‘rather moderate’. The students in all focus groups mentioned that the week of the course, which featured a very high workload, was highly exhausting.
5. Organizational aspects: Suggestions regarding changes to the group concepts focused on the time schedule (8/9) and the possible expansion of the self-experience group (9/9). The vast majority of participants in the relaxation groups would have preferred to have participated in the self-experience group. The question of whether such participation should be voluntary or compulsory was controversial. In particular, the participants in the self-experience group repeatedly claimed that they were grateful to have been assigned to this group because they would not have participated in it if they had been free to choose.
6. Difficulties with specific aspects of the intervention: In two groups, the process of working with the rank-dynamic model developed by Schindler was experienced as ‘difficult’ and ‘nonsensical’.

In the semistructured interview, the course leaders were asked about (1) observed changes during the intervention, (2) emotional closeness within the groups, (3) differences between the study groups, (4) problematic elements of the interventions and (5) adverse side effects. The themes for the thematic analysis were predetermined by the structure of the interviews.

1. Observed changes during the intervention: The self-experience group promoted cohesion in all groups and established an intensive relationship of trust within a short period. The experience of the universality of suffering, the support provided by fellow students and the process of taking time to examine one’s own goals were identified as particularly helpful.
2. Emotional closeness within the groups: Although the degree of openness varied from group to group, overall, the self-experience groups were characterized by a great deal of openness. Many personal experiences were shared, and the participants supported each other emotionally.
3. Differences between the study groups: The three therapists observed differences between the self-experience groups and the relaxation groups. Specifically, in the self-experience group, more personal development occurred, the participants appeared to be more energetic, active, and committed, more thought processes were triggered, and these processes had a more lasting effect than they did in the relaxation group. The participants often wanted to continue the sessions at the end of the self-experience group.
4. Problematic elements of the interventions: The therapists and the students themselves felt that it was important to pay attention to group dynamics. Working with Schindler’s rank dynamics model was experienced as problematic. Additionally, the students found it difficult to identify a concrete goal for change.
5. Adverse side effects: None of the adverse effects that were anticipated in this context, such as marginalization, breakdowns, breaches of confidentiality or discontinuation, were observed. However, students repeatedly approached the course leaders to ask where they could find help with perceived psychological difficulties. Although the students were free to determine the extent of their contributions, only one student in the relaxation group refused to participate.

## Discussion

The aim of this pilot study was to test the feasibility and effects of self-experience therapy during an internship in psychiatry. We hypothesized that short-term exposure to five sessions of self-experience training based on motivational interviewing would result in increased feelings of relatedness and intrinsic motivation and a reduced experience of stress compared with relaxation training.

Th**is** study revealed increased feelings of relatedness and intrinsic motivation among students in the self-experience group. While well-being and autonomous regulation increased in both study groups over the course of the internship, well-being increased slightly more in the self-experience group and just missed the significance level. No differences were found in terms of learning outcomes as measured by an OSCE. Overall, the self-experience training was very well received by the students. At the end of the semester, there were no differences between the study groups in terms of autonomous control, stress or well-being.

While self-experience therapy sessions are well established in the training of psychotherapists, no evidence or theory regarding the use of these sessions in medical education has been reported [25]. In line with our initial hypothesis, our self-experience group elicited more intense feelings of relatedness within the study group than the relaxation group did. This difference was quantified via the IMI and confirmed via focus group interviews with students and teachers. This finding is consistent with many other reports in the field of psychotherapy that suggest that group cohesion is one of the general, nonspecific factors in group therapy [52]. According to the students, this sense of relatedness led to greater intimacy throughout the practical psychiatry course, which reduced students’ anxiety while completing assessment tasks under the supervision of the entire group and increased students’ openness in the subsequent process of providing each other with feedback. In light of previous psychotherapy research, we decided to include an active control group (i.e., the relaxation group) to facilitate comparison with our self-experience group. Because psychotherapy, particularly group therapy, has many nonspecific effects, any intervention is nearly always better than doing nothing [53]. Consequently, the effect size of interventions always depends on the control group [53]. In our study, the difference between the two study groups was related mainly to the increased interaction with other participants observed in the self-experience group. In addition, the participants in the self-experience group were motivated to self-reflect and had the opportunity to share their feelings with other group members. Other nonspecific factors related to psychotherapy, such as taking care of oneself, being seen by others, acceptance, empathy and giving hope, were identical between these two groups [54].

Previous studies have reported that a sense of relatedness can be elicited by stimulating interaction among students [14,55]. Another study that involved a qualitative investigation of the effects of therapist-facilitated discussion groups in the context of surgery reported that the opportunity to reduce isolation and share struggles and thus establish a sense of community is very helpful in this context [56]. However, unlike the training of psychotherapists, medical students may know each other prior to such a course and may subsequently remain in contact. Therefore, the emergence of excessively intense intimacy in group discussions may lead to embarrassment. An opt-out model has been suggested as a way of offering mental health care services to health care professionals while simultaneously avoiding stigmatization [57]. In this context, the entire group is invited to participate, and each individual has the option to refuse. However, in our study, no students opted out. Not all participants completed the questionnaires, but all of them participated in the groups. Additionally, all students in the focus groups agreed that the level of intimacy that characterized these groups was appropriate. Students in several groups even suggested that the group sessions should be continued for an additional week, which was in line with feedback received for similar interventions [56]. Therefore, the relatedness and emotional closeness that evolved during the self-experience training was welcomed by the participating students and helped them build supportive relationships.

In line with self-determination theory, increased relatedness was accompanied by increased intrinsic motivation as measured by the interest/enjoyment subscale of the IMI [10]. Because no specific interventions were implemented for autonomy, competence or pressure, no differences were observed between the two groups with respect to these domains of the IMI. A large body of evidence links support for basic needs (autonomy, competence and relatedness) with intrinsic motivation and well-being [55,58]. However, Slemp et al. noted that most of the related data come from observational studies, making it difficult to draw conclusions about the direction of the relationships [55]. Although several studies have confirmed the assumptions of self-determination theory in randomized controlled trials with patients, very few such studies have been performed in the context of medical education [59,60]. Therefore, the results of this study support the assumptions of self-determination theory by adding experimental data in the context of medical education.

In addition to relatedness and intrinsic motivation, well-being increased in both groups over the course of the intervention. This finding is particularly encouraging because the proportion of students who received overall WHO-5 scores of 7 or less, which **is** highly indicative of a depressive episode, was 10.7% at the beginning of the course [47]. This proportion decreased to 3.3% following the interventions. While it is possible that this change was only partly the result of our interventions, a causal relationship seems likely. In particular, the course week is a week that features a very high workload, which elicits feelings of exhaustion at the end of each day. From Monday to Friday, the students spend four hours in the afternoon at the university in addition to their regular morning lectures. Nevertheless, their well-being increased. Since well-being is correlated with intrinsic motivation and the other subscores of the IMI, the observed trend toward increased well-being after self-experience was at least partly the result of the increased feeling of relatedness experienced by students in the group as well as the increase in students’ enjoyment during learning. A significant correlation between well-being, as assessed via the WHO-5 questionnaire, and intrinsic motivation has also been reported by other researchers [55,61]. Additionally, there is a large body of evidence linking relatedness in self-experience group therapy with well-being [25,62,63]. Therefore, it seems that motivational support can help students cope with heavy workloads while maintaining good mental health.

No differences were observed in the source of regulation between the two groups. In both the self-experience group and the relaxation group, autonomous regulation increased, whereas external regulation decreased over the five days of the course. The lack of differences in this regard is initially surprising because intrinsic motivation as measured via the IMI is moderately to highly correlated with autonomous regulation as measured by the SRQ. However, because this factor was measured at approximately six points on the seven-point Likert scale before the beginning of the course, the students who participated in this research nearly reached the limit of the SRQ. Consequently, the sensitivity of this measure to further differences was very low. Because autonomous regulation increased in both study groups during the course, it is possible that this change was not only a result of the interventions but also an effect of the bedside teaching included in the internship. Bedside teaching has previously been shown to increase autonomous regulation and decrease external regulation [64]. A conclusive effect of the self-experience group on the source of regulation was therefore not observed.

The specific effect of the relaxation group was a reduction in stress [20]. Several studies have reported that relaxation techniques can also reduce stress among medical students [21–23]. The lack of any increase in stress during the busy week of the psychiatry internship can therefore be attributed to the effects of the interventions. The self-experience group was no less effective than the relaxation group with respect to reducing stress. Since perceived stress is negatively correlated with intrinsic motivation and positively correlated with controlled regulation, it seems that the stress reduction observed in the self-experience group was caused by the promotion of intrinsic motivation in this group. This is of special interest because self-experience itself is likely to produce stress by provoking unpleasant feelings. In other words, although self-experience therapy is strenuous, it can reduce stress to the same extent as relaxation techniques by promoting intrinsic motivation.

It is initially discouraging that the effect of increased well-being among students in the self-experience group did not last until the end of the semester. Although the results of this pilot study suggest a promising way of reducing stress and increasing feelings of relatedness and intrinsic motivation for learning, several limitations of this research must be acknowledged.

First, the power of this trial, which featured 139 participants, was sufficient to detect only medium effects. Smaller effects may thus have been overlooked. Second, the dosage of the intervention was very low, i.e., only five sessions. Although these sessions were frequent, it is unlikely that their effects were long-lasting. For example, a meta-analysis of the effectiveness of Balint groups – groups of therapists who reflect together on a patient according to a predefined schedule with the aim of improving empathy – reported that the optimal number of sessions was ten [28]. Accordingly, no differences were observed between our two study groups at the end of the semester. This outcome can also be interpreted as confirmation that environmental changes, such as the introduction of a self-experience group into the curriculum, are more effective than individual interventions, which require behavioral changes by individuals within the group [24]. Moreover, sustained changes in the learning environment to support basic needs are likely to be successful in promoting students’ mental health [65]. Third, the extent to which this intervention is transferable to other contexts is uncertain. This was a single-center pilot study that involved students from only one semester. It remains to be seen whether the results can be reproduced in other cohorts in other university hospitals. Although both interventions are manual-based and can, in principle, be learned easily by experienced psychotherapists, therapists have a strong influence on the effectiveness of their therapies [54,66]. Researchers have even argued that the personalities of psychotherapists are several times more important than the methods that they use [67]. To avoid attrition effects in our study, all interventions were implemented by only three well-trained therapists. However, this makes the study susceptible to allegiance bias. Further research is needed to determine the extent to which this intervention is effective when implemented by other therapists. Finally, the students who participated in this study could not be blinded to their study group. In particular, because the self-experience group quickly gained a reputation for usefulness, the possibility cannot be ruled out that students in subsequent groups were biased in favor of the self-experience groups. **T**ogether with the self-reported nature of the questionnaires, this may have influenced the results. Given the high cost of the intervention (15% of curricular course time), the question arises whether the effects are clinically relevant. To assess this question, it is important to note that the effect of the self-experience group was tested against an active control group. Compared with other psychotherapy studies, the observed effect sizes for improvement in well-being (d = .261), relatedness (d = .505) or intrinsic motivation (d = .542) can be considered relevant [53]. Additionally, the reduction in the proportion of students at a high risk of depression from 10.7% to 3.3% in the pre- to post-intervention comparison suggests at least reasonable clinical relevance. Furthermore, the cost-benefit ratio improves when we note that the issues treated in self-experience group therapy are also learning goals of the German NKLM [30]. For example, graduates should be capable of introspection, self-reflection and communicating uncertainty.

Several strengths of this research should also be mentioned. Since this research featured a controlled longitudinal study design as well as an active control group, the level of evidence that it provides is quite high. The very high response rate of 83% for the entire semester cohort indicates that the results are representative of the entire cohort and allow valid conclusions to be drawn, at least for local students. The validity of the results of this research is also supported by its use of well-established questionnaires. Finally, the intervention tested in this study is theory-based; that is, it was developed on the basis of the established frameworks of self-determination theory and motivational interviewing. The results of this research, therefore, simultaneously test these theories and demonstrate their usefulness in the context of curriculum development.

## Conclusion

In this study, we presented the concept of a five-session, curriculum-integrated self-experience group to increase motivation and well-being among medical students and compared its effects with those of a relaxation group. This study revealed that such interventions are well accepted and can improve the well-being of medical students. Self-experience group sessions increase participants’ feelings of relatedness and intrinsic motivation in the short term, thereby reducing their perceived stress. However, the effects of the five sessions were very short-lived and wore off by the end of the semester. Elements of self-experience and interventions aimed at promoting emotional closeness in study groups are potentially effective ways to improve the mental health of medical students and should be more widely incorporated into curricula. Further research is needed to determine whether longer, ideally longitudinally integrated interventions lead to more sustainable effects.

## Data Availability

The data analyzed in this study can be made available by the authors upon reasonable request.
